# Consumers’ Intention to Adopt m-payment/m-banking: The Role of Their Financial Skills and Digital Literacy

**DOI:** 10.3389/fpsyg.2022.873708

**Published:** 2022-04-29

**Authors:** Saif Ullah, Umar Safdar Kiani, Basharat Raza, Abdullah Mustafa

**Affiliations:** ^1^National College of Business Administration and Economics, Lahore, Pakistan; ^2^Center for Entrepreneurial Development, Institute of Business Administration (IBA), Karachi, Pakistan

**Keywords:** financial skills, digital literacy, perceived usefulness, perceived ease of use, technology acceptance model, behavioral intention to adopt

## Abstract

The adoption of mobile payment (m-payment) and mobile banking (m-banking) is low in several countries, despite its associated benefits. The present study examines the impact of Pakistani consumers’ financial skills and digital literacy on their intention to adopt m-payment/m-banking using the Technology Acceptance Model (TAM). The data were collected from 454 individual smartphone users residing in Punjab province via an online and offline questionnaire survey. Structural equation modeling was used to analyze the consumers’ data. The results endorse that (1) their financial skills have no association with intention to adopt but through perceived usefulness; (2) their digital literacy bridges a strong association with intention and through perceived ease of use. Furthermore, this study discusses the theoretical and practical implications of the findings, as well as limitations and future directions.

## Introduction

Mobile-based payment services emerge as a leading digital platform with immense growth in mobile technologies, which facilitates financial transactions (Leong et al., 2021). People are now using different mobile services for communication and entertainment in general, as well as financial technology (Fintech) services (e.g., internet shopping, mobile payment systems, and transactions with the bank using smartphones) in particular. They prefer payment using mobile devices instead of traditional modes like cash, credit card, and debit card for acquired products (Carlsson et al., 2017). The mobile-based payments allow them to feel relaxed due to eliminating cash needs (Pham and Ho, 2015) coupled with speed and ease of use (Teo et al., 2015). These advantages have paved the way for the rise of mobile payments (Merritt, 2011) and many organizations are now aware of the importance of mobile payments (Andreev et al., 2011). The size of the global mobile payment market in 2019 was 1.48 trillion dollars, according to a report published by Allied Market Research in 2021. The report also predicts that the market will grow to 12.06 trillion dollars by 2027.

Mobile payment (m-payment) refers to a modern mobile telecommunication device through which financial payments are executed virtually by the general public, whether an internet connection is available or not. Among the other mobile-based services such as banking, commerce, internet banking, m-payment is a relatively new and underexplored research field (Oliveira et al., 2016). The acceptance of payment through mobile systems depends on various factors, including macro and micro-level factors. The macro-level factors include institutional support, technology access, and a feasible business model coupled with customer demand and a supportive business environment (Dahlberg et al., 2015; Evans and Pirchio, 2015; Arif et al., 2016; Liébana-Cabanillas et al., 2017). Some of the micro-level factors that have been studied earlier by various researchers affecting m-payment/m-banking adoption include trust, convenience, self-efficacy, subjective financial knowledge, mindfulness, innovativeness, perceived security, and perceived cost (Hanafizadeh et al., 2014; Al-Saedi et al., 2020; Alkhowaiter, 2020; Flavian et al., 2020; Li et al., 2020; Liébana-Cabanillas et al., 2020; Shankar and Rishi, 2020; Zhao et al., 2020). However, the findings of these studies have shown some inconsistencies related to study context, the number of respondents, and culture.

Despite the benefits and ease of use provided by associated technologies that fall under the mobile-based payment systems, adoption of these services is low in both developing and advanced countries (Lis and Kongaut, 2017; Kats, 2018; Zhang and Mao, 2020). In Pakistan, financial technology services were started around 2008, and from then a steady rise in new startups of various m-payment service providers offering financial assistance to the users. In December 2021, the number of cellular users in Pakistan reached a mark of 191 million with a teledensity of 86.71%, followed by the 3G/4G/Broadband subscribers (49.94% penetration) totaled 110 million (PTA, 2021). Despite the rising internet penetration rate, there is a scarce user base of m-payment and m-banking services. As per the State Bank of Pakistan, the total number of bank accounts reached 54.731 million out of the population of 204.65 million (as of 30 June 2019), indicating 26.74% of the population have a bank account (SBP, 2020). In the third quarter of the financial year 2020, the actual number of internet banking users and mobile phone banking users in Pakistan stood at 3.81 million and 8.18 million, respectively, whereas the total value of paper-based transactions for the third quarter of the financial year 2020 stood at PKR 32616 billion in comparison to internet banking and mobile phone banking transaction value of PKR 748.1 billion and PKR 467.5 billion, respectively (SBP, 2020). This indicates that people are more inclined towards paper-based cash transactions than internet-based and mobile phone-based banking transactions.

The m-payment/m-banking services adoption in Pakistan is relatively low compared to other developing countries such as India, Bangladesh, the Philippines, Indonesia, Thailand, etc. According to the Financial Access Survey (IMF, 2020) last updated on 15 October 2020, the registered figure for the mobile money accounts per 1,000 adults in Pakistan is 327.79 in 2019 which is far less than the other countries such as India 1264.79, Indonesia 1463.83, and Bangladesh 580.43. As for the number of depositors with commercial banks per 1000, Pakistan stood at 375.89, which is less than other countries including Bangladesh (792.01), the Philippines (658.28), Thailand (1,327.43), Turkey (1463.39), and Malaysia (705.23). In a nutshell, there is a huge economic potential that lies with the usage of m-payment/m-banking services as it would increase the consumer base as well as transaction volume in a country. So, it is necessary to determine what motivates people to use mobile payment/mobile banking services (Talwar et al., 2020; Zhao et al., 2020).

The present study contributes to the existing literature in two ways. First, the study investigates the direct and indirect effects of determinants like financial skills and digital literacy over m-payment/m-banking adoption intention using Technology Acceptance Model (TAM). Financial skill is about when and how to find, process, and execute financial decisions (CFPB, 2018). Whereas digital literacy means “*the awareness, attitude and ability of individuals to appropriately use digital tools and facilities to identify, access, manage, integrate, evaluate, analyze and synthesize digital resources, construct new knowledge, create media expressions, and communicate with others, in the context of specific life situations, in order to enable constructive social action; and to reflect upon this process”* (Martin, 2005, p. 135). The importance of skills and literacy has been acknowledged in numerous published reports and articles viz. CFPB (2018), LIRNEasia (2019), Sey and Hafkin (2019), UNESCO (2017); however, no study incorporates the role of financial skills and digital literacy as antecedents to m-payment/m-banking adoption intention regarding TAM. According to our knowledge, this is the first time the present study investigates factors like financial skills and digital literacy in the mobile payment and mobile banking field. Second, the study also presents a proposed integrated research model to improve our understanding of mobile payment/banking adoption intention, extending the TAM (Davis, 1989) by including determinants of adoption intention. The presumptions of the original TAM state the role of “perceived ease of use” and “perceived usefulness” as mediators between the external variables and intention to adopt the technology. The external variables were based on the characteristics of perceived behavioral control (financial skills and digital literacy), a construct from the Theory of Planned Behavior (TPB) (Ajzen, 1991). TPB states that attitude, subjective norms, and perceived behavioral control shape the intention of an individual to perform a specific behavior. According to one of the TPB presumptions, perceived behavioral control directly influences the behavioral intention of the individual. So, the study assumes that financial skill and digital literacy being the attributes of perceived behavioral control may influence the intention of the consumer in accepting mobile payment/banking services.

The following is the overall structure of this study. First, research hypotheses will be developed, along with a proposed research model, followed by a detailed methodology and measurements. Next, we detailed the results, followed by a discussion. Finally, we discuss the theoretical and practical implications of our findings, as well as their limitations and potential future research avenues.

## Literature Review

### Financial Skills and Behavioral Intention

It is noteworthy that researchers are currently looking into the critical factors that influence the adoption of technology systems, as reported by Yucel et al. (2013). In line with researchers, the present study uses perceived behavioral control (skills attribute) from the TPB and integrates it with TAM as an external variable. According to TPB (Ajzen, 1991), the perception of behavioral control (skills, for example) induces an individual’s intention to perform a behavior. Skill is an essential factor in accepting new and challenging tasks like online shopping (Novak et al., 2000). Because m-payments and m-banking involve financial transactions, the consumers of such services must learn and develop financial skills for better decision-making in funds management.

Financial skills are considered powerful abilities applicable to a wide range of financial decisions (CFPB, 2018). Financial skill is a necessary element of financial literacy. Financial literacy is concerned with an individual’s awareness of knowledge and applying that knowledge in formal and informal situations (Huston, 2010). The advancement of financial expertise has significant ramifications for the general welfare of the population (Lusardi and Mitchell, 2014). One factor contributing to the adoption of financial technology is financial education, also known as financial literacy. Higher financial literacy leads to a greater propensity to adopt financial technology because households with greater financial literacy can better understand Fintech services, including m-payment/m-banking (Jünger and Mietzner, 2020; Morgan and Trinh, 2020; Yoshino et al., 2020).

There is no denial of the importance of an individual’s financial knowledge in financial decision-making, but mere factual knowledge of finance is insufficient without acquiring a skill. Also, not every knowledgeable individual possesses the same level of financial expertise; individuals can grab these skills by observing the market, gaining hands-on experience in trade activities, and learning from their peers and colleagues. Financial skills include the techniques of planning one’s budget using mobile applications and understanding some banking basics, i.e., bank service fees, overdrawn fees, accessing one’s account, etc. (Take Charge America, 2017). A significant number of respondents believe that relying on “how to do things” rather than “knowing specific facts” is preferable in many situations (CFPB, 2015). According to previous research findings, mere factual financial knowledge is insufficient to effect behavioral change in an individual consumer (Ajzen et al., 2011; CFPB, 2015). It means that the role of financial skills is critical in understanding the usefulness of the technological product. It may lead to reshaping the consumer’s approach towards using technology like m-payment/m-banking services. Consequently, the study assumes that individuals who possess financial skills have a positive attitude towards the adoption of m-payment/m-banking services adoption.

**H1:** There is a positive association between financial skills and m-payment/m-banking adoption intention.

### Digital Literacy and Behavioral Intention

In the previous decade, the focus of extensive research was studying wireless internet technology, which resulted in various technology acceptance models grounded on TAM (Marangunić and Granić, 2015). These include acceptance of wireless internet (Lu et al., 2003; Wu et al., 2011) and acceptance of mobile internet (Lu et al., 2003; Son et al., 2012). In the past, several researchers emphasized the requirement for the inclusion of external variables in TAM for the better prediction of system use (Legris et al., 2003; Taherdoost and Masrom, 2009; Taherdoost et al., 2009) affirmed by Yucel et al. (2013). Thus, the determination of new and robust factors affecting the intention to adopt m-payment/m-banking will contribute to the broad knowledge base of m-payment/m-banking systems.

As advancement in the technological revolution highlights the importance of literacy like never before (Brown et al., 2005), digital literacy is essential to learning and experiencing new technologies. Its presence ensures the socio-economic development of citizens to become a part of modern and digital society to live and communicate better (Bejaković and Mrnjavac, 2020). It refers to *“the variety of literacies associated with the use of digital/new technologies”* (Mohammadyari and Singh, 2015, p. 10). Digital literacy is a modern and essential “life skill” acquired in the current information and knowledge society (Bawden, 2001; Markless et al., 2007). Individuals who want to use technology for day-to-day tasks are not using technology in a real sense (Yucel et al., 2013). Digital skills and access to technology are vital in exploring important untapped areas relating to learning and everyday life (UNESCO, 2017). Several prior studies reported that individuals who possess digital literacy use digital systems better than others. For example, Bergdahl et al. (2020), in their study, indicate that students who possess higher digital skill levels are more likely to get engaged in technology-enhanced learning systems. In another study, Ferro et al. (2011) reported that individuals having lower digital literacy levels do not prefer to engage in the decision to use web-based learning platforms.

Furthermore, a study by Darsono (2005) reported that computer-related self-efficacy has a direct positive impact on the intention to use technology. In the light of past research studies, the study assumes that individuals with higher digital literacy levels are more confident to use a digital platform like m-payment/m-banking systems, in line with TPB’s perceived behavioral control and intention relationship. Based on the above discussion, it is assumed that:

**H2:** There is a positive association between digital literacy and m-payment/m-banking adoption intention.

#### The Role of Perceived Usefulness as Mediator

One of the presumptions of TAM is the role of “perceived usefulness” as a mediator between the external variables and the intention to adopt the technology. In addition to the hypotheses mentioned earlier, the sequence of the study constructs displays that perceived usefulness serves as a mediator between the independent variable (financial skills) and behavioral intention to adopt m-payment/m-banking. Recently, a study conducted by Kirana and Havidz (2020) also suggests that advanced financial literacy and repeated use of mobile phones help increase financial inclusion. Data were obtained from 100 respondents via questionnaires and analyzed through linear regression followed by SPSS. The results indicate that financial knowledge and behavior along with perceived usefulness for m-payments have a positive effect on financial inclusion. Other studies also reported that individual behavior to use financial products largely depends upon one’s financial knowledge (Jünger and Mietzner, 2020; Morgan and Trinh, 2020). In the essence of prior studies, the present study establishes a link between financial skill and the financial service’s usefulness. The study argues that an individual who possesses financial skills is more aware of the usefulness of financial products than an individual who lacks these skills. Precisely, individuals with financial skills visualize more benefits of using an m-payment/m-banking system. Hence, the study assumes that financial skills induce the perceived usefulness of m-payment/m-banking services usage.

**H3:** Financial skills are positively associated with perceived usefulness.

Liébana-Cabanillas et al. (2020) found that perceived usefulness influences the intention to use mobile payment services. In another study, Shin et al. (2018) proposed an extended TAM model and concluded perceived usefulness impacts the intention of individuals directly and through attitude. Earlier studies also revealed that perceived usefulness is positively associated with attitude and usage intention (Davis, 1993; Hsu and Chiu, 2004; Liu et al., 2009; Liébana-Cabanillas et al., 2017). In line with the past studies’ results, the current study proposes that the perceived and imagined benefits of using m-payment/m-banking services positively induce an individual’s intention. When individual consumers believe that m-payment/m-banking is useful, they develop an intention to use these services to get the intended benefit. Moreover, TAM emphasizes that external variables may be responsible for a change in perceived usefulness, affecting an individual’s choice to adopt a technology. Therefore, our hypotheses are:

**H4:** There is a positive association between perceived usefulness and m-payment/m-banking adoption intention.

**H5:** Perceived usefulness mediates the association between financial skills and m-payment/m-banking adoption intention.

#### The Role of Perceived Ease of Use as Mediator

According to TAM, “perceived ease of use” served as a mediator between external variables and the adoption intention of the technology. PEOU is one of the TAM factors responsible for the individual user motivation towards using a particular system (Taherdoost, 2018). Previous studies have reported the direct relationship between digital literacy and individuals’ perception concerning its ease of use. For example, Ferro et al. (2011), in their study, indicate that individuals with low digital literacy levels do not engage in learning web-based technologies. It means individuals are not experiencing the ease of use due to weak foundations in digital literacy. In other words, individuals will perceive greater ease of use when they are digitally literate.

**H6:** Digital literacy is positively associated with PEOU.

Other studies also reported the substantial influence of PEOU on the adoption intention of mobile-related services (Keramati et al., 2012; Leong et al., 2013; Jaradat and Faqih, 2014). Earlier studies have established the association between PEOU, attitude, and usage intention (Schepers and Wetzels, 2007). However, scant research is available for PEOU and technology adoption intention reporting that there is no association between these two variables. For instance, a study conducted by Liébana-Cabanillas et al. (2018), targeting Spanish mobile users, reported that PEOU has no significant effect on the intention to use. Therefore, in this study, the relationship between digital literacy and intention to adopt m-payment/m-banking services via PEOU has been tested to gain more insights into m-payment/m-banking adoption intention.

**H7:** There is a positive association between PEOU and m-payment/m-banking adoption intention.

**H8:** PEOU mediates the association between digital literacy and m-payment/m-banking adoption intention.

**FIGURE 1:**
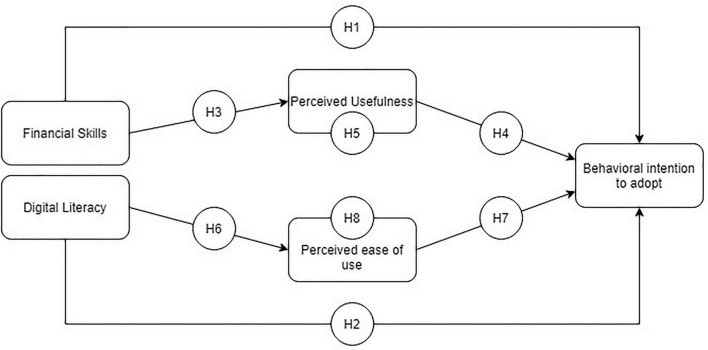
Research model and hypotheses.

The research model (Figure 1) is as follows:

## Methodology

### Participants and Procedure

The study uses a positivism research philosophy and deductive research approach to conduct quantitative research to examine consumers’ behavioral intentions regarding adopting m-payment/m-banking services. For this purpose, we used a survey research strategy, employing online and paper-and-pencil questionnaires to collect cross-sectional data from respondents (mobile consumers with broadband/3G/4G enabled services) residing in different cities across Punjab, Pakistan. The questionnaire was administered in both languages: English (original version) and Urdu (translated version). As the native language of the respondents is Urdu, therefore, those who can understand English were given an English version, and those who do not understand English were provided with an Urdu version survey. A back-translation method recommended by Bulmer and Warwick (1993) was employed. A linguistic expert with professional qualifications converted the English questionnaire into the Urdu language. The Urdu version of the questionnaire translates back into the English language to see if the translations were equivalent. The questionnaire contained two sections. The first section was about the basic demographic information of participants, while the second section incorporated the study variables-related statements and questions.

Moreover, we conducted a pilot test of the questionnaire to eliminate potential errors. Both the English and Urdu versions of questionnaires were pilot tested with sample sizes of 70 and 50 participants, respectively. The results of pilot testing confirmed that measures are valid and reliable for their intended purposes. Data collection took place in two-time waves with a gap of a 1-week interval to reduce the common bias (Podsakoff et al., 2012). A link to the online survey, created using Google forms, was sent to personal contacts via Whatsapp messenger service, with the request that they complete the form and also share the link with their contacts. We have received a total of 316 responses from the online administered questionnaire. We have also distributed paper-and-pencil surveys in both languages to random mobile consumers who found it convenient to read and understand the questionnaire content. Out of 300 questionnaires (150 of the English version plus 150 of the Urdu version), only 159 respondents returned the questionnaires (80 of the English version and 79 for the Urdu version), yielding a 53% response rate. After examining the data, 21 questionnaires (12 English and 9 Urdu versions) were discarded due to careless and incomplete responses, resulting in 138 usable questionnaires available for the final analysis (68 English and 70 Urdu versions). As per the rule of response per item (10:1), as suggested by Randall and Gibson (2013), the responses would be 330 per 33 items. However, the final sample consisted of 454 mobile users (online and paper-and-pencil respondents) to reduce the sampling error, non-responsiveness problems, and generalizability concerns (Bryman, 2016). The respondents’ sample includes employed, self-employed, unemployed, or students. It is also necessary to have student respondents (*N* = 162) because they possess tremendous potential and a strong desire to use mobile payment systems (Sohn and Kim, 2008).

### Measures

#### Financial Skill

Financial skill was measured in the first time wave, using a 10-item scale developed by Consumer CFPB (2018). This 10-item measure includes items like, “I know where to find the advice I need to make decisions involving money” and “I know when I do not have enough information to make a good decision involving my money.” The Cronbach’s alpha of this measure was 0.89.

#### Digital Literacy

Digital literacy was measured using a 10-item questionnaire developed by Ng (2012) on a 5-point Likert scale (“1 = strongly disagree,” “5 = strongly agree”) in the first time wave. Items include “I can learn new technologies easily” and “I have good ICT skills.” The internal consistency was 0.92.

#### Perceived Usefulness

Respondents completed the perceived usefulness measure (5-point Likert scale), adopted from Bhattacherjee (2001), Schierz et al. (2010) in the second time wave. The 4-item scale commences with an item, “The mobile payment/banking system is a useful mode of payment.” The other statements include, “Using a mobile payment/banking makes the handling of payments easier” and “I believe that a mobile payment/banking system improves my consumer decisions (providing flexibility, speed, etc.).” The scale anchoring is “1 = strongly disagree” and “5 = strongly agree.” The internal consistency was 0.94.

#### Perceived Ease of Use

The items in this scale are from Kapoor et al. (2015), Rana et al. (2014) measured on a 5-point Likert scale ranging “1 = strongly disagree” and “5 = strongly agree.” Participants responded with a reliability of 0.94. The items include “Mobile payments/banking are easy to use” and “Using mobile payments/banking easily for my things are important to me.”

#### Behavioral Intention to Adopt

The study adopts scale items from Bélanger and Carter (2008), Venkatesh et al. (2012), measuring intention to adopt on a 5-point Likert scale ranging from “1 = strongly disagree” and “5 = strongly agree.” The sample item is “I plan to use mobile payment/banking in the next months.” Participants responded with an internal consistency of 0.93.

The measurement items of study variables were presented in the Appendix (see Supplementary Material).

#### Control Variables

The study controls demographic variables such as age, gender, income level, and education. These variables may have significant influence over technology adoption as suggested by Morris and Venkatesh (2000); Venkatesh et al. (2000), and Lleras-Muney and Lichenberg (2002).

### Descriptive Statistics With Profile and Quality of Research Variables

Males (*n* = 311, 68.5%) exceeds the number females (*n* = 143, 31.5%) in terms of responses. The age bracket of most of the participants ranges from 20 years to 29 years, with the majority (*n* = 323, or 71.1%) falling between those ages. The majority of respondents (*n* = 205, 45.2%) have bachelor’s and master’s degrees (*n* = 147, 32.4%) education, with the remaining respondents having intermediate (*n* = 40, 8.8%) or advanced degrees (MS/M.Phil. and Ph. D., *n* = 49, 10.7%) education.

Table 1 includes the descriptive statistics, reliability, and correlation tests. The Harman single factor test method is used, which determines whether or not a factor exists (Podsakoff et al., 2003). This study employed the multi-wave response method (Podsakoff et al., 2012). In this study, a single latent variable loads into all scale items. It explains 43.6% of the total variance, which is less than the 50% threshold, indicating that common method bias is not a problem in this data (Mattila and Enz, 2002). There is an adequate internal consistency in all the study measures (α > 0.7) (Fornell and Larcker, 1981; Nunnally and Bernstein, 1994). In this study, the values representing the correlations among variables are less than 0.7, portraying no multicollinearity issue (Tabachnick and Fidell, 1996).

**TABLE 1:** Descriptive statistics, internal reliability, and correlation (*r*).

	1	2	3	4	5	6	7	8
1. Financial skill	(0.89)							
2. Digital literacy	0.566**	(0.92)						
3. Perceived ease of use (PEOU)	0.431**	0.631**	(0.94)					
4. Perceived usefulness	0.433**	0.602**	0.574**	(0.94)				
5. Behavioral intention to adopt	0.409**	0.547**	0.647**	0.687**	(0.93)			
6. Gender	−0.088	−0.089	−0.082	−0.066	−0.110*	NA		
7. Age	0.211**	0.200**	0.135**	0.167**	0.137**	−0.031	NA	
8. Education	0.100*	0.107*	0.000	0.064	0.055	0.126**	0.484**	NA
Mean	3.05	3.50	3.89	3.93	3.59	1.32	3.63	2.60
SD	0.82	0.88	1.04	1.03	1.01	0.48	1.20	1.05

**p ≤ 0.01, *p ≤ 0.05. The values reported in the parentheses forming diagonal represent Cronbach’s alpha (α). Gender: 1 = Male, 2 = Female. Age: 1 = Less than 15, 2 = 15–19, 3 = 20–24, 4 = 25–29, 5 = 30–34, 6 = 35–39, 7 = 40 and above, 8 = Prefer not to answer. Education: level 1 = Intermediate, 2 = Bachelor, 3 = Master, 4 = MS/M.Phil., 5 = Ph.D., 6 = Other.

As a surprise, financial skill positively linked to the behavioral intention to adopt (*r* = 0.41, *p* ≤ 0.01), PEOU (*r* = 0.43, *p* ≤ 0.01), and perceived usefulness (*r* = 0.43, *p* ≤ 0.01). Aside from that, it seems that digital literacy is positively associated with behavioral intention to adopt (*r* = 0.54, *p* ≤ 0.01), PEOU (*r* = 0.63, *p* ≤ 0.01) and perceived usefulness (*r* = 0.60, *p* ≤ 0.01), among other things. The PEOU (*r* = 0.64, *p* ≤ 0.01), as well as the perceived usefulness (*r* = 0.68, *p* ≤ 0.01), is associated with the behavioral intention to adopt. Furthermore, consumers’ gender correlates with their intention to adopt m-payment/m-banking consistent with the study of Glavee-Geo et al. (2017).

Anderson and Gerbing (1988) stated that a measurement model must be established before a structural model can be tested and validated, and the literature supports this practice. The results of the proposed 5-factor model are shown in Table 2, which indicates that the values of the fit parameters are satisfactory based on the data (χ2 = 1320.244, df = 475, χ2/df = 2.779, CFI = 0.93, NNFI = 0.92, RMSEA = 0.063) meeting the requirements of the recommended benchmark values (χ2/df < 3, CFI > 0.95, NNFI > 0.95, RMSEA < 0.08) (Bagozzi and Yi, 1988; Browne and Cudeck, 1993; Hu and Bentler, 1999). Moreover, the values less than 0.90 are deemed as satisfactory results for Comparative Fit Index (CFI) and a Non-Normed Fit Index (NNFI) (Cheung and Rensvold, 2002). According to Table 2, a 1-factor model depicts an insufficient fit for the data (χ2 = 5148.979, df = 492, χ2/df = 10.465, CFI = 0.619, NNFI = 0.591, and RMSEA = 0.145). We decided to keep the suggested five-factor model as a result of our findings because it provides the best fit values.

**TABLE 2:** Outcomes of confirmatory factor analyses (CFA).

Variables	χ2	Df	Ratio χ2/df	CFI	NNFI	RMSEA
1-factor model^a^	5148.979	492	10.465	0.619	0.591	0.145
2-factor model^b^	4275.967	491	8.709	0.691	0.667	0.130
3-factor model^c^	2393.179	484	4.945	0.844	0.830	0.093
4-factor model^d^	1797.078	479	3.752	0.892	0.881	0.078
5-factor model^e^	1320.244	475	2.779	0.931	0.923	0.063

*^a^“financial skills,” “digital literacy,” “PEOU,” “perceived usefulness,” and “behavioral intention to adopt,” all combined as single-factor.*

*^b^“financial skills,” “digital literacy,” “PEOU,” “perceived usefulness,” in a single factor, and “behavioral intention to adopt” in a single factor.*

*^c^“financial skills,” “digital literacy,” in a single factor, “PEOU” in a single factor, and a single factor each for both “perceived usefulness” and “behavioral intention to adopt”.*

*^d^“financial skills” in a single factor, “digital literacy” in a single factor, “PEOU,” “perceived usefulness” in a single factor, and “behavioral intention to adopt” in a single factor.*

*^e^“financial skills,” “digital literacy,” “PEOU,” “perceived usefulness,” and “behavioral intention to adopt” each, in a single factor.*

Table 3, confirms that the condition of convergent validity has been met in its entirety (AVE ≥ 0.50, CR values are more than 0.7 (Fornell and Larcker, 1981) with factor loading per item ≥ 0.5). Tables 1 and 3 demonstrate that the square root value of AVE is greater than the correlations between constructs, fulfilling the discriminant validity criteria (Zait and Bertea, 2011).

**TABLE 3:** Factor loadings and validity of scales.

Constructs	Construct items	Loadings	CR	AVE	√ AVE
Financial skills	FS1 FS2 FS3 FS4 FS5 FS6 FS7 FS8 FS9 FS10	0.721 0.764 0.807 0.719 0.675 0.745 0.676 0.616 0.627 0.500	0.897	0.500	0.707
Digital literacy	DL1 DL2 DL3 DL4 DL5 DL6 DL7 DL8 DL9 DL10	0.718 0.721 0.748 0.718 0.756 0.789 0.830 0.717 0.688 0.562	0.918	0.530	0.728
Perceived usefulness	PU1 PU2 PU3 PU4	0.875 0.551 0.828 0.576	0.941	0.799	0.894
Perceived ease of use	PEOU1 PEOU2 PEOU3 PEOU4	0.565 0.598 0.948 0.603	0.940	0.796	0.892
Behavioral intention to adopt	BI1 BI2 BI3 BI4 BI5	0.552 0.538 0.545 0.515 0.639	0.933	0.736	0.858

### Data Analysis

We tested our hypotheses using structural equation modeling (SEM), which we conducted using the Statistical Package for Social Sciences (SPSS 22) and the Analysis of Moment Structures (AMOS 22) software packages, respectively. We investigated the direct and indirect effect of mediation using a technique of bootstrapping (ten thousand samples along with 95% CI), and we found that it was significant (Iacobucci et al., 2007). The study used a structural model (SEM) for hypothesis analysis and testing. The findings demonstrate that the model met all of the criteria for being a good fit for the situation (χ2 = 1320.244, df = 475, χ2/df = 2.779, CFI = 0.93, NNFI = 0.92, RMSEA = 0.063).

## Results of the Research

The findings of our analyses and the hypotheses testing are available in Table 4. Evidence suggests that financial skill is associated with behavioral intention to adopt; however, the relationship is statistically insignificant (β = 0.080, *p* > 0.05). As a result, data do not support hypothesis 1. Specifically, the findings of this study support hypotheses 2 and 6: digital literacy is positively associated with behavioral intention to adopt (β = 0.140, *p* < 0.05), and the latter is linked directly with PEOU (β = 0.751, *p* < 0.05). Hypothesis 3 is supported (β = 0.547, *p* < 0.05) because financial skill is positively associated with perceived usefulness. According to our findings, perceived usefulness and behavioral intention to adopt were positively related (β = 0.404, *p* < 0.05), and PEOU and behavioral intention to adopt were both positively related (β = 0.217, *p* < 0.05), providing strong support for our 4 and 7 hypotheses.

**TABLE 4:** SEM results.

Study hypotheses	Paths (Hypothesized)	β	*t*-value	*P*-value
H1	FS→BI	0.080	1.199	0.071
H2	DL→BI	0.140	2.392	0.004*
H3	FS→PU	0.547	6.927	0.000***
H4	PU→BI	0.404	9.603	0.000***
H6	DL→PEOU	0.751	17.295	0.000***
H7	PEOU→BI	0.217	5.341	0.000***

***p ≤ 0.01, *p ≤ 0.05. FS = Financial skills; DL = Digital literacy; PU = Perceived usefulness; PEOU = Perceived ease of use; BI = Behavioral intention to adopt. Gray area in table represents significant values.

According to Table 5, the analysis supports Hypotheses 5 and 8. Perceived usefulness, as predicted, mediates the relationship between financial skill and behavioral intention to adopt. Increasing financial skill increases perceived usefulness, which increases the behavioral intention to adopt. Furthermore, PEOU mediates the relationship between digital literacy and behavioral intention to adopt because digital literacy increases PEOU, as well as behavioral intention to adopt.

**TABLE 5:** The mediating roles of perceived usefulness and perceived ease of use.

	95% BCa-CI			
	Estimate	Lower	Upper	*P*
Relation between FS and BI	0.08	−0.004	0.17	0.121
Mediating impact of PU between the relationship of FS and BI Relation between DL and BI Mediating impact of PEOU between the relationship of DL and BI	0.22 0.14 0.16	0.15 0.05 0.07	0.30 0.25 0.26	0.001 0.012 0.003

*BCa-CI: “Bias-corrected and accelerated bootstrapping confidence intervals.” Estimates grounded on ten thousand bootstrap samples. FS = Financial skills; DL = Digital literacy; PU = Perceived usefulness; PEOU = Perceived ease of use; BI = Behavioral intention to adopt. Gray area in table represents significant p-values.*

## Discussion

Grounded on TAM and TPB theory, this research study demonstrates whether the association between financial skills and digital literacy over the intention to adopt m-payment/m-banking is mediated by perceived usefulness and PEOU, respectively.

Results revealed no significant positive association emerged between financial skills and intention to adopt m-payment/m-banking, as anticipated in Hypothesis 1. This result is not in line with the previous research studies (Morgan and Trinh, 2020; Yoshino et al., 2020). This insignificant association indicates that there might be other factors responsible for our study hypotheses, including digital literacy, perceived usefulness, and PEOU inducing intention to adopt m-payment/m-banking. According to TPB, an individual’s activities relating to intention and subsequent behavior also depends upon various available means and prospects such as skill, time, cooperation received from others, etc. (Ajzen, 1991).

The results also showed that digital literacy has a significant positive impact on behavioral intention to adopt m-payment/m-banking of users, coherent with the past studies (Darsono, 2005; Bergdahl et al., 2020). And in line with TPB’s perceived behavioral control (skill) or “volitional control” influencing the individual’s intention, hence, supporting Hypothesis 2.

As expected, there is a significant positive relationship between financial skills and perceived usefulness supporting our Hypothesis 3. It means individuals who have higher financial skills perceived more usefulness related to a particular financial service. Furthermore, perceived usefulness also significantly influences users’ intention to adopt an m-payment/m-banking system. This result affirms the theoretical view presented in TAM (for a detailed theoretical view, see Davis, 1989) and is also in line with earlier studies (Liu et al., 2009; Shin et al., 2018; Liébana-Cabanillas et al., 2020), supporting our research Hypothesis 4. Results also revealed that perceived usefulness completely mediates the association between financial skills and m-payment/m-banking adoption intention (study Hypothesis 5). In brief, individuals possessing financial skills perceive more usefulness of m-payment/m-banking services by comparing its advantages (i.e., time, security, etc.) with other substitute services, which in turn, leads to an intended usage of m-payment systems. Moreover, this mediation showed a novel result in triggering individual users’ intention toward using m-payment/m-banking systems in the presence of basic financial skills.

Our results confirm Hypothesis 6, revealing that digital literacy positively influences PEOU attached to m-payment/m-banking systems. This result indicates that a person well-versed in digital literacy will experience more ease in using m-payment/m-banking systems. This result also supports TAM’s presumption that external variables might influence PEOU. Results also reflect that PEOU significantly impacts intention to use m-payment/m-banking, confirming study Hypothesis 7, in line with TAM’s postulate and prior research (Schepers and Wetzels, 2007; Taherdoost, 2018). Moreover, in line with Hypothesis 8, PEOU mediates the relationship between digital literacy and m-payment/m-banking adoption intention. This partial mediation result signifies the novelty of the relationship, in line with TAM’s assumption that PEOU mediates the link between external variables and intention to adopt the technology. Individuals with higher digital literacy levels perceive more ease in operating m-payment/m-banking applications resulting in induced intention to adopt these applications.

## Theoretical Implications

According to TAM, external variables may influence PEOU and perceived usefulness related to technology, which induces adoption intention for a specific technology. Original TAM does not discuss the direct impact of external variables on the intention to adopt the technology. Only two predictors affect intention to adopt technology named perceived usefulness and PEOU. This study integrates the volitional control or perceived behavioral control characteristics and TAM. Keeping in view the assumptions of TAM, the study results provide insights into the direct and indirect influence of Pakistani consumers’ financial skills and digital literacy on the m-payment/m-banking adoption intention. The empirical results showed that digital literacy affects m-payment/m-banking intention except for financial skills. This practical and novel validation of the direct effect of digital literacy on adoption intention contributes to the extant literature on TAM.

Furthermore, the study also observes the indirect effects of financial skills and digital literacy over intention supporting TAM assumptions. Results also indicate that the proposed research model is novel and uniquely extends TAM. Financial skills and digital literacy appeared to be antecedents of perceived usefulness and PEOU, respectively, in pursuit of determining external variables emphasized by many researchers (Legris et al., 2003; Taherdoost et al., 2009; Yucel et al., 2013).

This study reveals new horizons of investigations for prospective researchers concerning the knowledge and skill component of the individual in accepting m-payment/m-banking systems. Future studies may examine the role of financial skills and digital literacy in different context settings.

## Practical Implications

This study is the first endeavor to examine the role of individual users’ financial skills and digital literacy as external variables and TAM constructs. These variables are essential to mobile payment services providers as the intention of an individual to adopt m-payment/m-banking depends on the degree of change within them. There is a need to market m-payment services in such a fashion that promotes the learning and usage of these services smartly. The study results are also crucial for banks, retailers, IT experts, and Government to take essential steps to boost the financial inclusion drive in a country by focusing general publics’ financial ability and digital literacy skills through various platforms (offline and online). One of the online platforms is the use of social platforms like blogs, wikis, social media, web-based platforms, providing an opportunity for users to interact with each other by sharing experiences of m-payment (Miltgen et al., 2013). Using the social platform to induce m-payment/m-banking acceptance intention via learning and training-based advertisement may increase m-payment/m-banking adoption. Moreover, this practice will ensure user satisfaction and enables successive recommendation (Oliveira et al., 2016).

In addition, this study will help managers of Fintech service providers to revise and formulate new policies to attract prospective consumers of m-payment/m-banking systems. Present and future mobile phone users may also take benefit from this research by equipping themselves with the necessary financial skills and digital literacy to use mobile payment platforms along with traditional financial transactions. Furthermore, the current study helps strengthen the economy through digitalization and provides new opportunities to reduce unemployment by managing human resources. The understudied research also contributes to environmental challenges faced by the globe through the limited use of paper.

## Limitations and Future Avenues

Nevertheless, the study incorporates valuable and novel insights about the mobile-based payments adoption intention of individuals in the light of theoretical and practical contributions; it is not short of limitations. First, one limitation is the employment of cross-sectional data. The study analyzed data in two-time phases with 1 week gap to reduce common-method bias (Ployhart and Vandenberg, 2010). Second, the generalization of the study results is not possible concerning individuals residing in other countries. The data collection process took place in Pakistan and represents a small sample of the general public. Future researchers should consider cross-cultural settings to examine the role of financial skills and digital literacy on usage intention of different m-payment systems like m-wallet, NFC, QR code-based services. Third, the present research follows a quantitative study approach. Future studies may use a mixed-method strategy to examine consumers’ m-banking/m-payment behavioral intention adoption. Fourth, the focus of the current study is to look at behavioral intention to adopt m-payment/m-banking services rather than actual adoption. Future studies may examine the actual usage behavior of the consumers regarding m-payment/m-banking adoption.

## Conclusion

Based on TAM and TPB’s perceived behavioral construct characteristics, this study examines the association of consumer financial skills and digital literacy intending to adopt m-payment/m-banking services in Pakistan. Results indicate that perceived usefulness completely mediates the relationship between financial skills and intention to adopt, whereas PEOU partially mediates the association between digital literacy and intention to adopt. This study will assist the concerned stakeholders, including individuals, m-payment/m-banking service providers, and the Government, to take steps for individuals learning through market awareness programs, education, and training because these skills are essential for the benefit at large.

## Data Availability Statement

The raw data supporting the conclusions of this article will be made available by the authors, without undue reservation.

## Author Contributions

SU had been primarily initiated and completed the research work. All authors contributed to the article and approved the final version.

## Conflict of Interest

The authors declare that the research was conducted in the absence of any commercial or financial relationships that could be construed as a potential conflict of interest.

## Publisher’s Note

All claims expressed in this article are solely those of the authors and do not necessarily represent those of their affiliated organizations, or those of the publisher, the editors and the reviewers. Any product that may be evaluated in this article, or claim that may be made by its manufacturer, is not guaranteed or endorsed by the publisher.
