# Supplementary material for: Consumers’ Intention to Adopt m-payment/m-banking: The Role of Their Financial Skills and Digital Literacy
Source: Front Psychol. 2022 Apr 29;13:873708. doi: 10.3389/fpsyg.2022.873708 (PMC9100703; doi:10.3389/fpsyg.2022.873708)
Supplement: Supplementary file 1 [file Table_1.DOCX]

**Appendix**

**Measurement Items of study variables**

| **Construct** | **Items** |
| --- | --- |
| Financial skill | 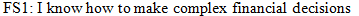 |
|  | 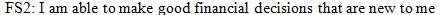 |
|  | 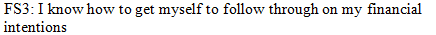 |
|  | 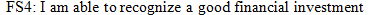 |
|  | 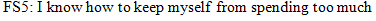 |
|  | 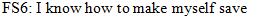 |
|  | 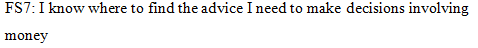 |
|  | 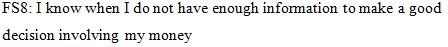 |
|  | 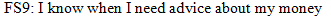 |
|  | 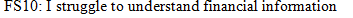® |
| Digital literacy | 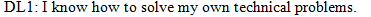 |
|  | 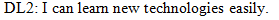 |
|  | 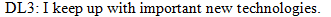 |
|  | 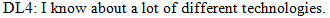 |
|  | 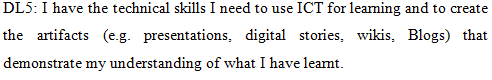 |
|  | 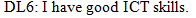 |
|  | 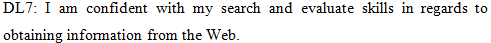 |
|  | 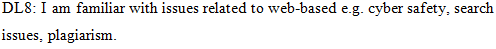 |
|  | 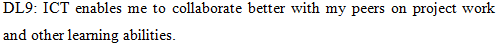 |
|  | 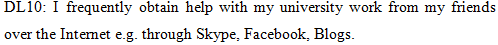 |
| Perceived ease of use | 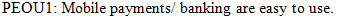 |
|  | 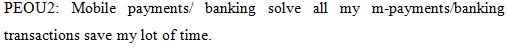 |
|  | 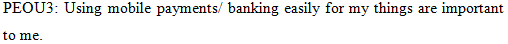 |
|  | 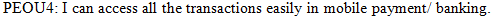 |
| Perceived usefulness | 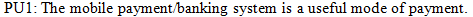 |
|  | 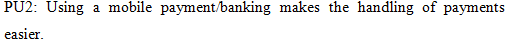 |
|  | 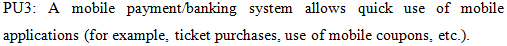 |
|  | 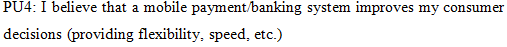 |
| Behavioral intention to adopt | 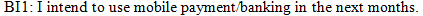 |
|  | 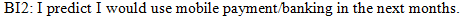 |
|  | 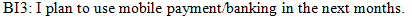 |
|  | 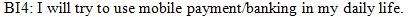 |
|  | 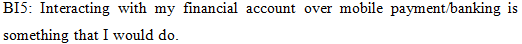 |
